# Supplementary material for: Can mutual health organisations influence the quality and the affordability of healthcare provision? The case of the Democratic Republic of Congo
Source: PLoS One. 2020 Apr 16;15(4):e0231660. doi: 10.1371/journal.pone.0231660 (PMC7162613; doi:10.1371/journal.pone.0231660)
Supplement: S1 File — (DOCX) [file pone.0231660.s001.docx]

## **Questionnaire gestionnaire/membres du Conseil d’Administration de la mutuelle de santé**

## Questionnaire Managers/Members of Board of Governors of the mutual health organization

**Descriptif général de la mutuelle de santé**

General description of the Mutual Health Organization

**Préparation et le processus de création**

The preparation and the creation process

**Structure organisationnelle, gouvernance, prise de décision**

Organizational structure, governance, decision-making

**Questions générales concernant la gestion de la mutuelle**

General questions concerning the management of the mutual health organization

**L’adhésion et la mobilisation de ressources**

Membership and resource mobilization

**Les services offerts par la mutuelle de santé**

Services offered by the mutual health organization

**Les relations avec l’offre de soins ; utilisation des soins**

Relations with the supply of care; utilization of care

**La gestion financière**

Financial management

**Les résultats de la mutuelle de santé: statistiques**

The results of the mutual health organization: statistics

## **Questionnaire prestataire**

## Provider questionnaire

**Les données chiffrées disponibles**

Available data

**La qualité des soins**

Quality of care

**Les contraintes liées à l’offre de services de qualité et les conditions nécessaires pour la maintenir ou l’améliorer**

The constraints linked to the supply of quality services and the conditions necessary to maintain or improve them

**La qualité de la gestion du système de santé**

The quality of health system management

**Opinion sur les prévisions de la mutuelle pour influencer la rationalisation de l’offre de soins**

Opinion on the mutual health organization forecasts to influence rationalization of care

**Y a-t-il une contribution de la mutuelle de santé à l’amélioration de la qualité de l’offre de soins ? Résultats concrets en termes de qualité de soins résultants de négociations avec la mutuelle, conditions du contrat, apport financier, etc. Expliquez.**

Is there a contribution from the mutual health organization to improve the quality of the care offer? Concrete results in terms of quality of care resulting from negotiations with the mutual, conditions of the contract, financial contribution, etc. Explain.

**Les relations mutuelle(s) – prestataire**

Relationship Mutual Health Organization(s) - provider

## **Questionnaire autorités sanitaires de la zone**

## Questionnaire health authorities in the area

**Données générales de la zone**

General data of the area

**Des statistiques de la zone de santé pour comparer à celles de la mutuelle**

Statistics of the health zone to compare with those of the mutual health organization

**La problématique de l’accès aux soins (de première et deuxième ligne) au niveau du système local de santé ; les barrières à l’accès aux soins**

The problem of access to care (first and second line) at the level of the local health system; barriers to access to care

**La qualité de l’offre des soins, aussi bien dans le secteur public que privé**

The quality of care provision, both in the public and private sectors

**Les contraintes liées à l’offre de services de qualité et le conditions nécessaires pour la maintenir ou l’améliorer**

Constraints related to the supply of quality services and the conditions necessary to maintain or improve them

**La perception de la qualité de cette offre aussi bien par les populations (côté de la demande) que par les prestataires (côté de l’offre)**

The perception of the quality of this offer by both population (demand side) and providers (supply side)

**La qualité de la gestion du système de santé**

The quality of health system management

## **Questionnaire structure d’appui local**

## Local support structure questionnaire

**Date de début des activités de la structure d’appui au niveau local**

Start date of activities of the support structure at local level

**De qui venait l’initiative ?**

Whose initiative was it ?

**Le rationnel de la création de l’organisation ; en réponse à quel(s) problème(s) a-t-elle été constituée**

The rationale for creating the organization; in response to which problem (s) was it created

**Les objectifs au niveau local**

Objectives at the local level

**Nombre de mutuelles appuyées + dates de démarrage**

Number of mutual health organizations supported + starting dates

**Description succincte du fonctionnement, monitoring & gouvernance de la mutuelle de santé**

Brief description of the operation, monitoring & governance of the Mutual Health Organization

**Les ressources financières et matérielles**

Financial and material resources

**Les ressources humaines**

Human resources

**Aperçu des activités hebdomadaires, occasionnelles**

Overview of weekly & occasional activities

**Interventions auprès des mutuelles de santé (nombre, objet)**

Interventions at the level of the mutual health organizations (number, subject)

**Interventions auprès des prestataires de soins**

Interventions at the level of the healthcare providers

**Capacité de répondre à la demande et/ou aux besoins**

Ability to meet demand and/or needs

**L’utilisation des mécanismes de monitorage et de suivi mis en place (utilisation du logiciel ou de registres pour un suivi manuel) : qualité du suivi**

The use of monitoring and follow-up mechanisms put in place (use of software or registers for manual monitoring): quality of monitoring

**Production de rapports mensuels/trimestriels/annuels**

Production of monthly/quarterly/annual reports

**Difficultés de gestion rencontrées**

Management difficulties encountered

**Limites de compétence ressenties**

Competency limits felt

**Opinion sur les résultats de chaque objectif de la structure d’appui**

Opinion on the results of each objective of the support structure

**Les conditions nécessaires pour réaliser les objectifs**

The conditions necessary to achieve the objectives

**Institutionnalisation et professionnalisation**

Institutionalization and professionalization

**Opinion en terme de la qualité et la rationalisation de l’offre des soins**

Opinion in terms of quality and rationalization of the care offer

**Suggestions pour étendre la couverture de la population et de services**

Suggestions for expanding population and service coverage

**Suggestions pour optimaliser l’appui aux mutuelles de santé**

Suggestions for optimizing support of the mutual health organization
