# Supplementary material for: Can mutual health organisations influence the quality and the affordability of healthcare provision? The case of the Democratic Republic of Congo
Source: PLoS One. 2020 Apr 16;15(4):e0231660. doi: 10.1371/journal.pone.0231660 (PMC7162613; doi:10.1371/journal.pone.0231660)
Supplement: S2 File — (DOCX) [file pone.0231660.s002.docx]

**Additional file 2: Summary of results per MHO**

**M1: Selection of providers**

| MUSOSA | - Urban citizens do not accept the poor quality of public health providers and want to be seen by a medical doctor. Providers are selected by the MHO among preferred private medical centres and hospitals. - Poor people travel to close-by rural public health facilities that receive substantial international subsidies. |
| --- | --- |
| KLA | - Health facilities of the public health system: public, integrated faith-based and private |
| MUSSRA | - Health facilities of the public health system: public, integrated faith-based and private |
| Nyantende | - No real choice in rural areas - > Public (mainly integrated BDOM) health structures in their health area |
| Walungu | - No real choice in rural areas -> Public health structures in their health area: Public hospital + health centres where at least 15 households of the health area are member |
| South Kivu overall | - Quality is not an issue mentioned in relation to choice. When quality is discussed, it is financial issues that come up, e.g. the financial integrity of various actors. |
| Kinshasa overall | - There are few health centres without medical doctor; health facilities are medicalised, with the exception of the BDOM. - There are few quality healthcare providers that have accessible fees. - Private providers come and go, without much control by the health authorities. |
| Lisanga | - Based on people’s preferences: ‘street interview’ at the start of the programme. - No government first line health facilities in the health zone. |
| MESP | - Selection by MESP on basis of location of members; mainly health facilities of the public health system: public, integrated faith-based and private. |
| MUSECCO | - Health facilities of the public health system: public, integrated faith-based and private |
| UMUSAC | - Health facilities of the public health system: public, integrated faith-based and private. Each MHO of the UMUSAC network cooperates with the health facilities of their area. |

**M1: Members’ choice among providers**

| MUSOSA | - Free choice among contracted health facilities. 50% attend one hospital |
| --- | --- |
| Goma (MUSSRA & KLA) | - Members must register with a contracted health centre or medical centre in their area (in line with pyramidal healthcare system). They are allowed to change once if they move or are not happy with the services. Perceived quality is the main reason for change. |
| KLA | - Members must register with a contracted health centre or medical centre in their area but in practice many members do not observe this rule. - Without subsidies, most first line health facilities cannot offer quality care and members seek a better provider. |
| MUSSRA | - Members’ perception that more expensive care equals higher quality care influences their choice of provider. - Many members sought providers who would collaborate in arrangements for the financial benefit of both that often involved fraud. |
| Nyantende | - Members can go to all contracted health facilities in the province. - When ill, most people first turn to faith healers. They often only seek modern health care when seriously ill. Fees for faith and traditional healers can be much higher than a health centre (2$) or hospital (6 et 8 $) consultation. |
| Walungu | - Members can go to all contracted health facilities in the province. - Most people attend faith-healers and private unskilled providers because of affordability and drug stock-outs in health centres. |
| South Kivu overall | - The ‘choice’ is currently with the provider: providers tend to refuse members from MHOs that are insolvable. |
| Kinshasa overall | - Financial exploitation of patients is the rule. “Elsewhere” will not be better. |
| Lisanga | - People can chose between the contracted health facilities - Exit of members does not influence quality because of small numbers. |
| MESP | - Members must register with a contracted health centre or medical centre in their area. |
| MUSECCO | - Members must attend the health centres and hospitals closest to their homes. |
| UMUSAC |  |

**Exit from the MHO**

| All MHOs | - The package of services covered is the most frequent reason for drop-out: e.g. decreased financial coverage; hospitalisation in common wards; too many services to be paid out-of-pocket, etc. These issues, caused by financial limitations, have nothing to do with clinical care quality. However, the high fees charged by the providers, compared to the limited resources of the MHO, are direct cause for this decrease of benefits. |
| --- | --- |
| MUSOSA | - Many members are very loyal since the start. However the benefits have become insignificant and members threaten to leave the MHO. |
| KLA | - Affiliation and drop-out are mostly related to individual financial benefits of the members (mainly adverse selection). |
| MUSSRA | - Affiliation and drop-out are related to individual financial benefits of the members (adverse selection; cheap access to expensive treatments; possibilities for fraud). |
| Nyantende | - Drop-outs related to decreased financial benefits (in 2016: coverage decreased from 80% to 60%); drop in membership when the NGO Maltezer stopped paying premium of 1444 poor people. - Care quality was only mentioned in relation with its fees. |
| Walungu | - Drop-outs related to decreased financial benefits offered by the MHO; drop-out once ailment treated; drop-out when the person/organisation who paid premiums stops doing so. |
| South Kivu overall | - Important drop-outs in all MHOs with the switch from reimbursement of 80% to 60%; substantial drop in membership each time an international partner stops subsidizing membership. Clinical quality not mentioned as a factor. |
| Kinshasa overall | - The turnover of members in the MHOs of the CGAT network is high. Poor patient reception and care quality explain most drop-outs. “Voting with their feet” is therefore at the level of the MHO that is punished where quality of the contracted providers is poor. |
| Lisanga | - The first health facilities that were evaluated were government facilities. There were no medicines and members were not satisfied. Now, 80% of health facilities are private. Satisfaction with services is above 70% (which does not mean that quality is optimal). -> Exit from the MHO influences choice of providers by MHO. |
| MESP | - Not applicable: contribution is automatically deducted from salary. |
| MUSECCO | - No quality-related drop-outs. Membership was stable and increasing until the creation of the MESP. |
| UMUSAC | - Drop-outs related to ability to pay and perceived benefits compared to practices of auto-medication and preference of traditional healers. |

**M2—Co-producing a long voice route: Schemes strategically purchase health care from providers, which gives a mandate to set quality standards**

**Selection of providers: Enlisting providers that meet defined standards**

| MUSOSA | - Quality assessment done at the start. - 12 providers: hospitals and medical centres; 3 government, 8 faith-based, 1 private. |
| --- | --- |
| Goma (KLA & MUSSRA) | - Formal CGAT quality assessment done. First line facilities with insufficient score can still be included but are asked to make changes. Improvement is followed but sometimes disappointing. |
| KLA | - See Goma - 14 providers among which 1 hospital (poorer area of the city) |
| MUSSRA | - See Goma - 8 providers among which 3 hospitals (richer area of the city) |
| South Kivu overall | - All public health facilities are included; mostly of the BDOM network. No quality assessment done. |
| Nyantende | - See South Kivu overall - 1 BDOM hospital, 10 health centres (2 government, 8 faith-based), 1 private medical centre. |
| Walungu | - See South Kivu overall - 1 public hospital and 4 health centres currently included |
| Kinshasa overall |  |
| Lisanga | - Formal CGAT quality assessment done. - The cost of care is too high in most health facilities. The network of the Salvation Army was not selected because too expensive (one exception). - Selection alone is not enough to guarantee quality: work on quality and cost-containment must follow. - 38 first line (of which 7 private-for-profit, 3 public, 25 BDOM, 3 other faith-based); 2 referral hospitals |
| MESP | - Formal quality assessment done; technical advisor mandated by the provincial health inspector. - 90 health facilities: 1^st^, 2^nd^ and 3^rd^ line; specific services outsourced to specialised health facilities. - During the first year all health facilities of BDOM and Salvation Army were included. Later, the number was reduced to make follow-up easier. Therefore, many members do not any longer attend the health centre of their health area. Some health centres lose their clients while others have a high workload. |
| MUSECCO | - Quality assessment done in 1999 by ILO/STEP. - 68 health facilities: 57 BDOM, 8 Salvation Army, 2 Baptiste Community in Congo (CBCO), 1 government |
| UMUSAC | - Selection by medical advisor on basis of the quality approval assessments done by the health zone. - In addition, UMUSAC has its own health centre. |

**Establishing a contract with providers**

| All MHOs | - A contract is signed with all providers. It is seen as an important working document by all partners. |
| --- | --- |
| MUSOSA | - The contract is important to determine the MHO-provider relationship, but it is currently not respected because tariffs are outdated. Preparations for new tariffs underway. |
| Goma (KLA & MUSSRA) | - Model CGAT contract annually revised. The contract is very important to determine the MHO-provider relationship. It is the basis for settling any conflict. |
| KLA | - Respected, but agreeing on fees is an annual discussion. |
| MUSSRA | - See Goma |
| South Kivu overall | - Contracts are annually revised. Annual revisions are coordinated by the medical advisor. CAMS and MHO co-sign. |
| Nyantende | - See South Kivu overall |
| Walungu | - Contract with hospital: contract for this year not yet signed because of disagreement on tariffs. - One contract with the health zone for the health centres. |
| Kinshasa overall |  |
| Lisanga | - Contract with each provider, adapted from a template - BDOM coordinator signs 1 contract for all health facilities - In some health facilities: slow but progressive effect of the contract on the quality/expenses equation, or rather, of the regular discussion of quality and fees between medical advisor and providers. - In most: clauses concerning care quality are often not respected and difficult to inforce. |
| MESP | - Contract is signed with each provider after negotiation with public health authorities (general secretariat) or BDOM and Salvation Army for the faith-based sector. - Its main influence lays in its clear determination of services covered. |
| MUSECCO | - Contract with all providers; one contract with BDOM for all their health facilities. - “The contract is respected. One example: at one point when we found that the deficit was too important, we wrote to the MHO that we would break the contract at the end of the month, but were reminded the contract stipulates this should be announced three months in advance. It brought us around a table to harmonize our views.” |
| UMUSAC | - Each MHO has a contract with its providers. - Overall respected in health centres because it adopts the package of services and rules of the public health services. |

**Availability of medicines**

| MUSOSA | - Essential medicines are available in pharmaceutical warehouses. Most health facilities do not have sufficient resources to ensure availability of medicines. Hospitals with a large clientele of members benefit from the MHO to buy medicines. |
| --- | --- |
| Goma (KLA & MUSSRA) | - Essential medicines are available in pharmaceutical warehouses. Health facilities have difficulties keeping sufficient stocks. - When signing the contract the CGAT insist on drug availability. Because of frequent stock-outs, the clause is discussed in every meeting with providers. - It is not the contract that makes the difference but the MHO payments. |
| KLA | - Health centres do not have the resources to make monthly provisions. They purchase small quantities in private pharmacies. - The revenue from the MHO helps some providers to buy medicines. There are stock-outs when MHO payment is delayed. |
| MUSSRA | - See Goma |
| South Kivu | - Essential medicines are available in pharmaceutical warehouses (e.g. ASRAMES). Most health facilities do not have the resources to buy them. In most cases it is not the contract that makes the difference but the MHO payments. |
| Nyantende | - Frequent stock-outs in hospitals and health centres. For the hospital, the revenue from the MHO helps to buy medicines. |
| Walungu | - For the hospital availability of medicines is a priority. - The health zone does not have the resources to buy safe drugs for the health centres. Health centres are usually out of stock or buy small quantities on the black market. |
| Kinshasa overall | - Essential medicines usually available in health facilities. |
| Lisanga | In one clinic:   - Essential medicines always available; - Negative influence of the MHO: delay in payment for non-generic drugs causes stock outs -> members are given a prescription to buy outside   Overall:   - Several health facilities are frequently out of stock. Members can be reimbursed when they present an invoice from the pharmacy. It is a cause of dissatisfaction. - No real influence of the MHO on availability of medicines. The MHO is considering to have its own pharmacy. |
| MESP | - The contract stipulates that generic medicines must be available at the health facility. In case of stock-outs, it is up to the provider to buy them elsewhere. - In case of stock-outs of non-generic medicines, members may be given a prescription. MESP established a contract with one pharmacy for that purpose, where members can obtain them after control by a medical advisor. - Complaints about unavailability of medicines concern less than 5% of cases. |
| MUSECCO | - Essential drugs are usually available. It is a priority for the BDOM and for any health facility that wants to maintain a reputation of providing quality services. |
| UMUSAC | - Essential medicines are available in the city, less so in the peri-urban area. Laboratory reagents are often lacking. - No influence by the MHO. |

**Services covered**

| MUSOSA | - Clear list of services covered and proportion paid by the MHO. - The package is respected where the invoicing of the MHO is concerned, but the patients pay out-of-pocket what is not covered. - In one hospital prescribers do not know who is member, because they think all patients should be treated equally. - The list of services covered does not influence quality or prescription. |
| --- | --- |
| Goma (KLA & MUSSRA) | - Because patients’ fees are their only or main income, health facilities tend to provide and invoice services that are not covered or even not needed. The list of included services does not change prescription, but is the basis to refuse payment for invoiced services. |
| KLA | - Clear list of services covered. Few laboratory tests covered. - The hospital respects the list of services covered. |
| MUSSRA | - Clear list of services covered. Many services are excluded; few laboratory tests covered. Does not reduce huge over-prescription. |
| Nyantende | - Clear list of services covered. Many services are excluded, especially laboratory tests. - The list of services covered does not influence prescription. - The hospital tends to overprescribe, provide and/or invoice services that are not covered or even not needed, invoiced to MHO or patient. - Health centres tend to retain patients instead of referring to the hospital for financial gain. |
| Walungu | - Clear list of services covered. The proportion paid by the MHO changed from 80% to 60%, which confuses members. - The list of services covered does not influence quality or prescription: the package is respected where the invoicing of the MHO is concerned, but the patients pay out-of-pocket what is not covered. |
| Kinshasa overall |  |
| Lisanga | - Clear and comprehensive package of services, although many lab exams are excluded because expensive - Providers start to understand the rules after about 6 months. Members always want more which still demands much explanation and discussion with providers and MHO. - Providers can ask approval of the medical advisor for services outside the package, which is often granted. |
| MESP | - Clear and comprehensive package of services covered, including specific examinations and treatments for which approval by the medical advisor must first be asked. - It is strictly observed. - The strict control avoids that first line health facilities retain patients to treat them locally instead of referring. |
| MUSECCO | - Clear list of covered and excluded services; in line with the pyramidal structure of public health services. This package is always available and if not, immediately reported by the members. - Co-payment for first line is a fixed amount of 1000 Fc. - Financial coverage for child birth and surgery reduced in July 2016 to 80%. |
| UMUSAC | - Covered services are those in line with the basic package of public healthcare provision. It is respected by providers and members. |

**Gatekeeping for hospital care**

| MUSOSA | - No gatekeeping: no contracts with first line health facilities, in accordance with people’s preferences |
| --- | --- |
| Goma (KLA & MUSSRA) | - Strict rules for gatekeeping. Members often try to circumvent the rule but have to pay the hospital or reimburse the MHO when it is found that they received hospital care without referral from first line. |
| KLA | - See Goma |
| MUSSRA | - See Goma |
| Nyantende | - Strict gatekeeping rules that are respected. |
| Walungu | - Strict gatekeeping rules that are respected. |
| Kinshasa overall |  |
| Lisanga | - First line health facilities respect gatekeeping; medical centres can provide most services and rarely refer. - Members try to circumvent the rule. Hospitals do not pay attention and send the bill, but the MHOs ask the members who went to the hospital without referral to pay back. The rule is more and more understood and respected. |
| MESP | - Strict gatekeeping, except for emergencies. - Referral for second line services needs the approval of a medical advisor who will decide to which health facility the patient should be referred according to the specific needs. - In case of emergencies MESP has to be informed within 48 hours and is seen by the medical advisor. |
| MUSECCO | - Gatekeeping is respected; for emergencies, regularisation always follows the next day. |
| UMUSAC | - Strict gatekeeping rules that are respected, except for emergencies. |

**Respect of agreed tariffs and OOP payments**

| MUSOSA | - Agreed fixed tariffs set in 2010 and not changed since are no longer respected. The MHO is still invoiced the same amount, but the patient pays the difference. New tariffs are being determined. |
| --- | --- |
| Goma (KLA & MUSSRA) | - Fixed tariffs for a list of specific health services that include service fee, generic medicines and standard investigations. Financial coverage by the MHO is 90%. Non-included investigations and non-generic medicines may be included on a fee-for-services basis when the medical advisor agrees. Financial coverage is 50%. - Every year, when renewing the contract, setting tariffs demands a lot of discussion. Once agreed, tariffs are generally respected because of close supervision by the medical advisor. |
| KLA | - See Goma |
| MUSSRA | - See Goma - Many attempts to overcharge the MHO. The list of services and tariffs serves as a basis for discussing and reviewing invoices. |
| South Kivu overall | - Annually revised tariffs for each health facility. Since January 2016, financial coverage decreased from 80% to 60%. - Because hospitals are self-financing and many individual patients never pay their bill, there is a huge tendency to overcharge MHOs. Overcharging as well as invoicing for services not provided is frequent. |
| Nyantende | - Health centres respect agreed tariffs when invoicing the MHO, but the patients pay the difference. - Hospital management finds that CAMS imposes tariffs that are too low. Tariffs are often not respected. The all-inclusive fixed fee for a service or procedure is considered as a minimum fee, and any additional material or medicine is invoiced separately. The MHO management suspects that the hospital adapts its monthly invoice on basis of an average amount the MHO will be able to pay rather than on the provided services only. Also a considerable amount is charged to the patient. |
| Walungu | - Clear list of all-inclusive fixed fees for a list of conditions; pricelist for other accepted treatments, tests and medicines. - Health centres often do not respect tariffs. - There is no agreement yet between hospital and CAMS for this year’s tariffs. The hospital disputes the tariffs CAMS proposes. Last year’s tariffs are respected, but separate charging of ‘other accepted treatments, tests and medicines’ hugely inflates the invoice. Also the amounts charged to the patients are high. - According to the officer in charge of the health zone, the hospital overcharges. |
| Kinshasa overall |  |
| Lisanga | - Tariffs are negotiated with each health facility. - All-inclusive fixed tariffs per type of service: curative consultation, hospitalisation, standard imaging (echo and basic RX), transfusion, minor, medium and 2 interventions of major surgery, MCH (flat fee for 4 antenatal visits, 1 echo, birth, vaccination, pre-school consultation until 5). - MHO covers 90% - Not included exams and procedures are paid on a fee-for-service basis. When accepted by the medical advisor: MHO pays 50% and patient 50%. - First line: At first, all-inclusive fees are seen as a minimum fee to which procedures, examinations and medicines are added. Revision of invoices according to the contract is accepted, but seen as “discount fees”, which affects members’ reception. Some health facilities start to understand and improve members’ reception. - Referral hospitals do not respect agreed tariffs. They want to impose the fees they charge to enterprises. Until now the CGAT accepts their conditions and tries to work it out with the regulator (without success). |
| MESP | - For first line: all-inclusive fixed tariff per type of service: antenatal care, early childcare, child birth, minor surgery, curative consultation. Fixed fees are respected, but a supplement can be paid when the health facility’s expenses were exceptionally high. - Preferential tariffs were obtained for first line health facilities. Negotiation and setting clear tariffs is difficult with large hospitals “who do not need you”. This is one of the reasons why MESP contracted specialised medical centres for specific second line care, with whom clear tariffs could be agreed (e.g. 1 for medical imaging, 1 for ear-nose-throat, 3 for spectacles, 1 for physiotherapy, 1 for mental health). |
| MUSECCO | - Preferential all-inclusive fixed fees per type of service; negotiated with BDOM coordination for its network. Agreed fees are respected: until July 2016 the MHO paid by capitation calculated on basis of the agreed fees (see payment of providers). |
| UMUSAC | - Same tariffs as non-members; the MHO covers 50%. - Official tariffs are respected in health centres. (↔ The UMUSAC health centre was created to deal with over-charging by some providers) (?) - The referral hospital tends to over-charge MHO members. |

**Rules for use of generic and non-generic medicines**

| MUSOSA | - The clause on primary use of generic drugs is not respected. The MHO has no longer a medical advisor who could enforce the clause. Non-medical MHO staff has no authority on the subject. Non-generic drugs are a huge expense for the MHO. - Most medical doctors prescribe non-generic drugs. Patients demand to change the prescription when generics are prescribed. There is much resistance to antibiotics due to self-medication, which requires second line drugs. - Specialists automatically prescribe non-generic drugs. - Within the BDOM, frequent discussion with providers does not change prescription. The BDOM wants to organise training on rational prescription and generic drugs with authoritative external trainers. |
| --- | --- |
| Goma (KLA & MUSSRA) | - Non-generic medicines can be prescribed after approval of medical advisor. - Specialists cannot be convinced to prescribe generic drugs. “It is a terrible fight. They say we want to dictate how they should treat patients”. |
| KLA | - Every month there are about 300 prescriptions for non-generic medicines (for on average 611 episodes). - Members demand non-generics and are not well informed about generic drugs. |
| MUSSRA | - People want non-generic drugs. Prescribers convince patients that non-generic drugs are better, even when the MHO tells differently. - Non-generic drugs are prescribed to inflate the bill. - When providers invoice non-generic drugs without approval of the medical advisor, MUSSRA does not pay. - MUSSRA does not accept prescriptions to buy non-generic medicines outside the hospital to avoid financial arrangements between prescriber and local pharmacy. - Specialists cannot be convinced to prescribe generics. |
| South Kivu overall | - The contract specifies that generic medicines should be used, but more specialised treatments, diagnostics tests and medicines that are not covered by the all-inclusive fixed fees can be prescribed when necessary, without explicit permission of the medical advisor. - The clause in the contract serves as a basis to deny payment and revise the invoice. |
| Nyantende | - People accept generics. - The possibility to prescribe specialised treatments, diagnostics tests and medicines when necessary is used to inflate the bill. |
| Walungu | - The possibility to prescribe specialised treatments, diagnostics tests and medicines when necessary is used to inflate the bill. - Health centres may only prescribe generics and if otherwise the MHO does not pay. |
| Kinshasa overall |  |
| Lisanga | - Generics as a rule, included in the fixed fee; non-generic drugs after approval by the medical adviser. - CGAT uses the list of 140 medicines recommended by WHO, but some providers consider some of these as non-essential and invoice accordingly. - Many first line health facilities routinely use generics, but specialists in medical centres (e.g. neurology, ophthalmology, ENT, dermatologists) only prescribe non-generics. - Providers have to agree with revised invoices because of the contract, but there is no decrease yet in prescription of non-generic drugs. - The MHO ran out of cash before the end of the year because of the cost of non-generic drugs. |
| MESP | - Generic medicines are the rule. Non-generic medicines need approval of a medical advisor. - Specialised investigations need approval of a medical advisor. Patients are referred to specific clinics to avoid unnecessary prescription and improve quality (e.g. interpretation of imaging by specialists). - These rules are strictly observed in first line and for outsourced care; little control in large hospitals. |
| MUSECCO | - List of covered drugs according to the ‘WHO essential medicines adopted in the DRC’. - Generics are widespread and rarely pose problems. - Not clear whether non-generic drugs are nonetheless prescribed: “When medical centres give prescriptions instead of providing the medicines, the MHO has difficulties to reimburse products bought in pharmacies”. |
| UMUSAC | - Generic medicines are the rule in the MHO like in the public health system. - No problems reported. |

**Respect of (national) treatment guidelines**

| MUSOSA | - There are guidelines in the health facilities, but they are not respected. Designing, enforcing and controlling use of guidelines is the role of the health authorities. - The rural health zones supported by the European Development Fund (EDF) have detailed protocols that are respected. |
| --- | --- |
| Goma overall | - Public and integrated health centres have and respect national treatment flowcharts. - National guidelines are developed for some specific conditions. Some hospitals develop their own guidelines, others do not impose guidelines. The provincial authorities organise workshops to evolve towards standardised guidelines. |
| KLA | - See Goma |
| MUSSRA | - See Goma |
| South Kivu overall | - Because health facilities are self-financing, healthcare is heavily commercialised; guidelines aiming at rational prescription are usually not observed. |
| Nyantende | - Health centres overall respect national treatment flowcharts, although they do not always transfer when needed. - The hospital designed guidelines (with Medics Without Vacations) that health staff and prescribers respect according to the hospital manager. |
| Walungu | - Health centres have but do not respect national guidelines. - The hospital designed guidelines (with Medics Without Vacations) that doctors respect according to the hospital manager. |
| Kinshasa overall |  |
| Lisanga | - There are national guidelines for some diseases and flowchart for first line, but they are usually not respected. “It creates difficulties with each invoice validation.” - The CGAT does not have therapeutic guidelines. “Our guide is the contract. The providers prescribe, and we pay those services that are included in the package.” - The MHO does not have an influence on prescription. |
| MESP | - First line health centres generally observe therapeutic guidelines, controlled by BDOM or health authorities. - National guidelines for HIV, tuberculosis and vaccination are observed. - Local health authorities, BDOM and MHO have no influence on prescription by medical doctors, especially in the large hospitals. - The BDOM has treatment guidelines for medical centres, organises regular training and supervision, but the influence of pharmaceutical traders is strong. |
| MUSECCO | - Under the capitation system, the MHO did not follow the use of therapeutic guides. - Health centres have guidelines provided by the BDOM or health authorities. |
| UMUSAC | - First line health facilities routinely respect national treatment flowcharts. - National treatment flowcharts are strictly observed in the UMUSAC health centre. |

**Procedures for payment of providers**

| MUSOSA | - Monthly payment of invoices according to services and tariffs in contract - Control of entitlement by MHO management - Timely payments |
| --- | --- |
| Goma overall | - Monthly payment of invoices according to services and tariffs in contract - Control of invoice: of entitlement by MHO management; of services by medical advisor - Invoicing, controls and payment often suffer delays. - Payment by cheque to avoid handling cash |
| KLA | - See Goma - Monthly third party payment of providers; reimbursement to members for emergency services elsewhere and for non-generic medicines bought in pharmacy |
| MUSSRA | - See Goma |
| South Kivu overall | - Monthly payment of invoices according to services and tariffs in contract - Control of invoice: of entitlement by MHO management; of entitlement and services by medical advisor, not on-site but in the CAMS office in Bukavu - Invoicing, controls and payment often suffer delays. - Payment by cheque to avoid handling cash |
| Nyantende | - Health centres, that need the income to cover the month’s expenses, do not appreciate the delay between issuing the invoice and its payment. This influences members’ reception. - “Invoices are received and sent to Bukavu the 10th-15th, analysed by the medical advisor, the cheques are signed about the 25th-30th and distributed”. |
| Walungu | - Frequent delays in invoicing by the health centres lead to delays in payment which in turn cause unfriendly reception of members. - The cost of getting the invoices in Bukavu increases time between reception of invoice and payment of dues. |
| Kinshasa overall |  |
| Lisanga | - Monthly payment of invoices according to services and tariffs in contract. - The revision of invoices is the most important tool of the MHO to decrease expenses for health care. The time it takes causes delay in payment. |
| MESP | - Monthly payment of invoices according to services and tariffs in contract. - The MESP delegate in the health facility controls entitlement, checks whether the invoice contains required information, compares invoice with patient registers, checks whether invoiced services are provided, before transmitting the invoice to the medical advisor. |
| MUSECCO | - Until June 2016, monthly payment in a system of capitation: the MHO advanced a sum of $2.30 per registered person per month to the coordinator of the network health facilities, who distributed to the concerned health facilities according to the agreed fees. Control of providers’ invoices is done at the level of their coordination. - From July 2016 onwards: switch to fee-for-services payment because, according to the MHO management, the MHO pays more than the provided services. Controlling invoices becomes the role of the medical advisor of the MHO. |
| UMUSAC | - Capitation system in certain MHOs (monthly anticipated payment). Other MHOs pay fee-for-services after validation of the monthly invoice by the medical advisor. - MHOs using capitation will soon switch to fee-for-services payment in order to avoid to pay a higher price than needed. |

**Control medical advisor**

| MUSOSA | - Previously, a medical advisor controlled invoices and discussed his observations with providers. Currently the MHO management checks entitlement, respect of billing procedures and tariffs. They do not comment on prescription, because their authority in this field is not accepted. - All partners agree that a medical advisor is needed to avoid abuses, limit expenses and ensure guidelines are followed. The CGAT medical advisor, located in Beni, and does not travel to Butembo to take on this task. |
| --- | --- |
| Goma overall | - Validation of invoices:   - Monthly on-site check of invoice, registers and patients’ files:     - Invoice corresponds with treatment received     - Diagnostic tests and treatment are appropriate     - Invoiced treatments are covered by the MHO.   - Discussion and revision of invoice with accountant, management or health staff as needed.   - The contract is the basis to settle arguments. - Checking is very meticulous and item per item. When in doubt, received care is verified with the patient. - “There are very frequent attempts to invoice services that patients did not receive.” “For example, we received an invoice for 1900 $ but after validation it was reduced to 1200 $, a difference of 700 $ for one hospital for one month”. - Proportion of revised invoices: “All monthly invoices need one or another revision where the contract is not respected. So that makes 100%.” - Other roles of the medical advisor: Occasional check of overall care quality, settling problems with providers or patients. |
| KLA | - See Goma - Because hospital providers know that the medical advisor will refuse to pay non-generic medicines, they give a prescription and the patient has to buy the medicine privately. |
| MUSSRA | - See Goma |
| South Kivu overall | - The validation of invoices is done in the CAMS office in Bukavu. CAMS does not have the resources to pay for field visits by the medical advisors. They make one visit per health facility per year, for renewing the contract. Last year there were 168 health facilities for two medical advisors. - Monthly invoice sent by provider to MHO. A summary fact sheet is attached for each patient. The medical advisor checks invoice and fact sheets for congruence with the contract. - Providers rarely seem to dispute the proposed revisions after analysis. |
| Nyantende | - Perception that the medical advisor “imposes a way of working, controls the work of others, may dispute prescription and invoicing in order to reduce payment”. - The medical advisor notes the reasons for reduction of the invoiced sums in their report. - All invoices are reduced; sometimes with substantial reductions (see table)   **Nyantende: Difference between initial and revised invoice**   \| Month \| cases \| Amount invoiced \| After revision \| Difference \| \| --- \| --- \| --- \| --- \| --- \| \| Jan \| 109 \| 3619.48 \| 3073 \| 546.48 \| \| Feb \| 126 \| 4208.32 \| 3853 \| 355.32 \| \| Mar \| 121 \| 3712.52 \| 3631 \| 81.52 \| \| April \| 147 \| 3765.5 \| 3532 \| 233.5 \| \| May \| 147 \| 4033.85 \|  \|  \| \|  \| 650 \| 19339.67 \| 14089 \| 1216.82 \|  - Because medical advisors do not work onsite, they may miss the difference between hat is written in the sent documents and the reality (see citation).   « Mais le médecin conseil fait les révisions à partir des documents qu’il reçoit. Il y a ce qu’il ne voit pas. Je vous donne un exemple. Quand ils voient que tous les accouchements ont été eutociques, et qu’ils n’auront presque rien à la fin du mois, on peut ajouter des choses qu’on n’a pas faites pour expliquer le surplus du montant qu’on demande. Comment nous le savons ? Quand on montre la facture à la dame, elle se plaint qu’on ne lui a pas donné ce soin : « Je suis arrivée et j’ai mis au monde sans problème. Comment ils peuvent dire que j’ai eu ceci et cela. » Mais quand on pose alors la question, ils nous disent : « Ah, elle ne peut pas savoir. » On laisse comme ça, on envoie la facture. Moi, j’annexe une petite note sur la fiche et je dis voilà ce malade n’a pas reçu ceci et cela. Mais sur la facture de ce membre on montre que le malade a eu tout cela. (NyMut) |
| Walungu | - Previously, the medical advisors did the monthly validation in the hospital and revisions were discussed. Later, written feedback was sent, which is now discontinued. - Hospital management complains of lack of feedback about revised invoices: “The contract stipulates that if an invoice is revised, the medical advisor gives feedback. We await explanations for 1362 $ of unpaid debts”. - Because the medical advisor only sees the invoice and the attached patient files, the MHO management attaches observations to the invoice to point out over-charging or incorrect billing, especially for the health centres. |
| Kinshasa overall |  |
| Lisanga | - 4 medical advisors for 9 MHOs in the CGAT network. - Monthly control of invoices by medical advisor. For BDOM: combined monthly invoice for the 25 health centres, with summary for each patient (complaints, diagnosis, exams, treatment) annexed. On-site verification if necessary. For other providers: on-site verification, medical advisor has access to the patient files, revisions are directly discussed with the provider. - The contract is the guideline for revisions. “We only apply what the contract describes.” - 9/10 if not 10/10 of monthly invoices need revision. |
| MESP | - 4 medical advisors. - The proportion of services rejected for payment has substantially decreased since providers get used to the system. Total downward revision of invoiced was less than 1% in the first half of 2016. |
| MUSECCO | - 1 medical advisor appointed by the Ministry of health to accompany, advise and promote MHOs. His role in validation of invoices had not yet started at the time of the data collection. - The BDOM had designed its own system to control entitlement and invoicing of members of MUSECCO (receipts signed by provider and patient, with patient’s telephone number). |
| UMUSAC | - The medical advisor is appointed by the Minister of health. - Monthly control of invoices is done on-site together with the provider. Any non-conformity of invoice with rules of the contract are directly discussed. There are rarely problems. |

**Quality control by health authorities**

| MUSOSA | - The underfunded health zone has little capacity to control or improve care quality - Recently, the district health authorities participated in calculating average fees, and want to be involved in promoting rational prescription to improve accessibility of health care services. |
| --- | --- |
| Goma overall |  |
| KLA | - The health zone does monthly supervisions of hospital and health centres but has limited capacity to influence care quality. |
| MUSSRA | - The health zone does not intervene to promote rational prescription; is absent as regulator. |
| South Kivu overall |  |
| Nyantende | - The health zone does monthly supervisions of hospital and health centres. They have an influence on care quality, especially when medicines are available in their depot. |
| Walungu | - The health zone does monthly supervisions of health centres, but has little influence on prescription and respect of guidelines. |
| Kinshasa overall |  |
| Lisanga | - The relatively well-functioning health facilities have frequent controls by the health zone. For others the under-funded health zones have little capacity to intervene. |
| MESP | - Overall, underfunded health zones have little capacity to control or influence care quality. - The BDOM continues to oversee and support its 60 health facilities. Their main concern is to continue to provide quality medicines. Each health facility is self-financing. |
| MUSECCO | See MESP |
| UMUSAC | - Clear implication of the health zone in quality control of services. |

**Contribution of MHO to quality?**

| All MHOs | - Availability of medicines is seen as the most important factor determining quality care. From the users’ perspective, being well received and, in cities, being seen by a medical doctor, comes next. Clinical quality is rarely mentioned. Most questions about quality of care are answered with remarks about the cost of care: for the providers, a concern about the difficulty to provide care without sufficient income; for the MHOs, a concern about the unaffordable fees asked for health care. |
| --- | --- |
| MUSOSA | - Providers do not see care quality as a matter for the MHO, especially not without medical advisor. Controlling quality is the role of the health authorities. - “The MHO does not have the authority to discuss care quality and fees with the providers. You need the backing of the health authorities, who should provide treatment guidelines and promote affordable pricing.” - “Also the BDOM has lost its capacity to control care quality and tariffs, since it stopped providing medicines to the health facilities of its network.” - “Neither MHO nor BDOM have much say. Only the health authorities can impose standards and their implementation.” |
| Goma overall | - The MHO helps to improve care quality in the sense that it improves cost-recovery. That is why hospital managers start to appreciate the MHO. - The authority of the medical advisor: “Maybe for MHOs with high membership, the medical advisor may have a voice, but only if he has the official backing of the health authorities. Care quality is the responsibility of the health authorities, who can impose rules and sanction when they are not followed.” - The controls by the medical advisor have a small and slow influence on prescription, treatment and pricing. - “Care quality is the responsibility of the health authorities. The controls done by the medical advisor are to check whether the clauses of the contract are respected. This difference is fundamental. When the MHO demands rational prescription and generic medicines, physicians think that we judge their performance, which they do not accept. Especially specialists refuse our intervention. To make this difference between validating in accordance to the contract and quality control, we need the cooperation of the health authorities, who also should perform their part of quality control.” |
| KLA | - Health centres benefit in areas with large membership. Regular controls by the medical advisor help to improve care quality. - Positive influence in one health centre: The CGAT co-financed the rehabilitation of the health centre that now attracts patients again. Income increased. The reliable income from the MHO helps to buy medicines. Health centre and MHO cooperate well and the nurse in-charge promotes MHO membership. - The hospital manager sees no impact on care quality: the MHO pays the bills but the number of members is low. - Reception of members starts to be threatened by difficulties to pay providers. |
| MUSSRA | - Care quality was discussed in terms of rational prescription only. - Frequent downward revision of invoices has a negative effect on members’ reception. |
| South Kivu overall | - Whenever care quality was discussed with any interviewee in South Kivu, this was understood as the capacity to pay providers who then can provide care. - Overall, providers still consider the MHOs more creditworthy than the general population. However, due to the chronic imbalance between the modest premium paid by members and the much higher expenses for health care, most MHOs are not able to pay their bills, which affects members’ reception. Increasingly, health facilities refuse any further cooperation with insolvent MHOs. - Aspects of clinical quality were very rarely mentioned. |
| Nyantende | - “A large proportion of the hospital’s revenues comes from the MHO. It allows us to buy medicines and pay staff, and thus participates in a sense to care quality.” - For the health centres, the responsibility for care quality is clearly put with the health authorities. - MHO leaders comment quality in terms of rational prescription only, not in terms of clinical quality. |
| Walungu | - Clinical quality is not seen as a responsibility of the MHO. - “Care quality in the hospital is good because of a partnership with Medics Without Vacations. The only concern is drug procurement.” - “Instead of contributing to care quality, with its unpaid bills the MHO endangers quality of care in the hospital”. - “There are no visits of the MHO or CAMS to control and discuss care quality in the health facilities”. - “When the MHO is not able to honour its debts, this affects reception of members because the health facilities are self-financing.” |
| Kinshasa overall | All MHOs of the CGAT network:   - Selection of providers is the most effective way to improve care quality for members.   - “The first health facilities were government facilities, but there were no medicines and members were not satisfied. Now, 80% of health facilities are private. Satisfaction with services is above 70%”.   - However: “There are few health facilities that provide affordable quality care”; “It is difficult to find health facilities where patients are not financially exploited. Over-prescription is the rule.” - At the start of a new MHO-provider cooperation, patient reception is a problem, but this changes over time. This evolution is attributed to the regular discussions with the medical advisors and in the forum for dialogue. Currently, the MHOs are appreciated by the contracted providers. Over-prescription remains a problem. - “Care quality is the most important problem for the MHOs. The contracts are not applied, which makes unsatisfied members leave the MHO. In order to improve services, an organised forum for discussion is the solution. This sustained concertation where MHO and providers meet will in time solve the problem of responsiveness to people’s needs.”   -> Not the contract and its rules as such, but the exchange between medical advisor and health staff; between MHO and providers influences quality. |
| Lisanga | See Kinshasa overall.  Example of good cooperation with a medical centre:   - Renowned for good quality - Staff: 22 medical doctors: 5 generalists, 16 specialists; 30 nurses. - 70 % of patients from specific enterprises with whom the clinic has a contract - 20 % of patients are from the MHO, “we would like more”. - Good understanding between both parties. - Advantages for the clinic are that “the MHO keeps their promises for about 95%, do a good control of care quality and utilisation, and there is a lot of interaction with the medical advisor”. - The 3-monthly meeting is mentioned among the positives. - Among the negatives: - Members often try to obtain more than what is covered. They also try to abuse the system for internal referral. They ask the generalist to prescribe a referral to one or more of the clinic’s specialists for the same day. For the billing, this is considered as one consultation. Without referral, the patient pays a second consultation. - At the start, members received both generics and non-generics at the clinic. Delay in payment for non-generics caused stock outs -> members are given a prescription to buy outside. - Dilemma: “we know the services covered and the rules for prescription, but we lose when prescribing according to the agreement”. |
| MESP | - According to the MESP, the outsourcing of certain investigations and treatments improves the quality of these services. According to the BDOM, the inconvenience for the patient affects quality and continuity of care. - According to the BDOM, feedback from MHOs helps to improve quality for issues that had escaped their attention (e.g. reception, drug use, rational prescription). - All other quality improvements mentioned are related to the regular income from the MESP, especially in first line health facilities where many MESP members attend. Some health facilities were able to invest in infrastructure and equipment which in turn attracts more clients. - Apart from secured income, the health facility staff does not observe any influence of the MESP on care quality. “It is not because there is an MHO that we should provide better care.” - The main measure affecting clinical quality is the selection of providers. |
| MUSECCO | - According to the BDOM, feedback from MHOs helps to improve quality for issues that had escaped their attention (e.g. reception, drug use, rational prescription). - All other comments link quality with the income from the MHO. Overall, providers like MHOs because of the stable income (unless/until they go bankrupt), but they consider that with MUSECCO they lose out:   - “MHO members are welcomed and treated with care. No health facility wants to lose an MHO member because compared to individual patients who often cannot pay for their care, revenue from the MHO is secured.”   - “With enterprises who pay for their staff, we have a profit margin. But for MUSECCO, we work at a loss: the fixed fee of $ 7 comprising laboratory and medicines does not cover the services provided.” – “It is not MUSECCO that allows us to sustain drug supplies.”   - “Over the years, it is the BDOM that supported MUSECCO. Capitation payment was calculated on basis of one episode per person per year. In reality, their utilisation is 2.5 episodes per year. Other MHOs pay according to provided services, unless they abandon us.” |
| UMUSAC | - The regular income from MHOs helps providers to improve care quality. Providers seek to attract and retain MHOs because this ensures an increased clientele. Members’ reception is therefore good. The establishment of a contract motivates both providers and MHOs to respect their mutual engagements. - Nevertheless, UMUSAC found it useful to create its own health centre. Explanations for this apparent contradiction were not sought during the interviews. |

**Effectiveness of cost-containment measures**

1. Contextual factor: self-financing of health structures leads to over-prescription, over-charging and fraud
2. Capacity to promote cost-containment.

**Self-financing of health structures leads to over-prescription, over-charging and fraud**

| MUSOSA | - The public health facilities have little government support other than the modest salary or bonus for staff. For faith-based and private health facilities, patients’ fees are the only income. - “As long as the health authorities do not provide financial support, hospitals are not able to reduce fees.” - “On the one hand, health facilities have no other income than patient fees, on the other hand, MHOs seek affordable quality care. This creates a conflict.” - The district authorities describe the difference between the heavily subsidised health services in rural areas and the self-financing urban health facilities. In the rural health zones, adherence to treatment guidelines is part of the contract and a condition to obtain subsidies. In the urban zones, hospitals over-charge those who can pay in order to function. The district authorities are now cooperating with the MHO to study whether and how MHO’s and providers’ financial needs can be harmonised. |
| --- | --- |
| Goma overall | - “Self-financing leads to overbilling or unnecessary investigations that inflate the bill.” - “Without government subsidies, hospitals and individual physicians seek income elsewhere. When marketing their products, pharmaceutical firms provide a substantial percentage to the prescriber. Physicians also make arrangements for specialised examinations with private clinics. This does not promote rational prescription.” |
| KLA | - “The public sector is totally underfunded. Health staff is demoralised, which really affects care quality.” - Faith-based referral hospital: “The health authorities appoint some staff members, but do not pay their salaries. The government imposes free treatment for malnutrition and tuberculosis, but does not provide subsidies. Only some vertical programmes such as family planning and VIH receive support from international organisations.” - “The population if the zone is poor. Cost recovery in the hospital is low.” |
| MUSSRA | - “Hospitals entirely depend on patients’ fees. They will always maximise income to the detriment of patients.” |
| South Kivu overall | - “There should be government subsidies to pay for salaries. With a decent salary secured, MHOs could negotiate more successfully to obtain quality care at affordable prices”. |
| Nyantende | - “The hospitals entirely depends on patients’ fees”. - “The fact that salaries of health personnel are not paid by the government pushes towards self-financing and overcharging.” |
| Walungu | - Two hospital physicians have a decent salary paid by the government, the others, more recently engaged, do not. Other hospital and health centre staff receive a very low remuneration. International organisations occasionally provide drug donations. All other expenses are to be covered by patient fees. |
| Kinshasa overall | - Overall difficulties for the health facilities that do not receive financial support - Overall context of looking for individual financial gain - Providers tend to think that MHOs have lots of money, so they tend to over-prescribe and write invoices when no services were provided. - “Medical doctors act like supermarkets. They have no ethical standards.” |
| Lisanga | See Kinshasa overall |
| MESP | “The enemy of the MHO is fraud. The hospital is a business.” |
| MUSECCO |  |
| UMUSAC |  |

**Effectiveness of cost-containment measures**

| MUSOSA | - No influence on rational prescription. Patients pay out-of-pocket what the MHO does not pay. Discussing rational prescription is the role of the health authorities. |
| --- | --- |
| Goma overall | - “The MHO continuously discusses evidence of over-prescription, over-charging and fraud, but does not have the weight to influence these behaviours. Hospital managers want to cooperate with the MHOs, but medical staff tends to seek personal financial benefits.” - “The problems of over-charging should be solved by the health authorities. However, nearly all personnel of the health zone also works in the health facilities. It is difficult to point at irregularities where you work. Hospital management would say: “if we implement that, how will we pay you?”” - “The CGAT would like to have their own medical centre, where essential medicines would be available and qualified staff could treat members according to rational treatment guidelines.” |
| KLA | - The hospital management claim they already provide low-cost care because they serve a poor population; they respect the tariffs and conditions of the MHO contract. - The proportion of prescriptions for non-generic medicines remains very high. |
| MUSSRA | - The providers consider the MHO as a source of income. Over-prescription, over-billing and fraud are substantial. Detecting and minimising fraud is a time-consuming occupation of MHO manager and medical advisor. This tight control reduces the invoice, but does not yet change providers’ behaviour in a positive way. - “Providers are convinced the MHO is financed by external donors. When the MHO cannot pay their bills any longer, they think it is because we stole these subsidies. However with repeated explaining, some start to understand that services are paid with members’ contributions”. |
| South Kivu overall | - No cost-containment, to the contrary: - Most providers consider the MHOs as a cash cow and inflate the invoice. - Providers are convinced that external grands are the MHOs main source of income. Even if often discussed, they do not believe that services are solely paid with members’ contributions. Most believe that if the MHO asks for cost containment, it is for the enrichment of the MHO leaders. - “Misuse of funds is a fact of our society and therefore also of the MHO system. All parties will try to benefit. The mechanisms that limit opportunities for fraud must be strong. …”. “The other problem is of course embezzlement from all sides, and its normalisation in people’s values.” - The control by the medical advisor does reduce the invoice for the MHO, because it detects attempts to over-invoice. It does not influence rational prescription. Because the medical advisors do not control onsite, much of the over-invoicing escapes them. |
| Nyantende | - The revision by the medical advisor substantially reduces the invoice, but this does not influence prescription and billing behaviour. |
| Walungu | - The MHO management tries to avoid adverse selection, discusses rational prescription with members, made great efforts to find external subsidies, but does not take action to influence prescription: “the problem is that the medical advisor does not visit the providers”. |
| Kinshasa overall | All MHOs of the CGAT network:   - The exchange between medical advisors and providers has an effect: “progressively, providers understand that one can provide quality care with a lot less expenses”. Nevertheless, understanding does not equal changing behaviour: “For the providers, money is the priority. They act more like shopkeeper than like health practitioners who should be at the service of patients. They constantly look for opportunities to overcharge or abuse the system.” - First line: the bill is reduced by the revision of invoices, but there is little influence on overall prescription behaviour; no influence on prescription of specialists. - No influence on hospital bills. - CGAT contacts regulator for affordable fees in referral hospitals, without success. - “The regulator does not supervise quality and fees.” - The MHO needs the health authorities for control and enforcement of prescription guidelines and fees. |
| Lisanga | See Kinshasa overall |
| MESP | - Over-prescription, over-billing and fraud are a huge problem that needs constant “combatting” by strict rules and tight supervision by the 4 medical advisors and the MESP delegates in the health facilities. “Take away the delegates and you will see what happens.” - Consumption of medical imagery and specialised investigation has decreased since they are outsourced. Invoicing of non-provided services dramatically decreased since the introduction of delegates. A study will be done to evaluate the effectiveness of outsourcing and working with delegates. - As a more long-term measure, overconsumption is discussed with members, the effects on their health as well as the financial repercussions for the MHO. - No influence in large hospitals. - The BDOM that has its own system for cost-containment, does not approve of the outsourcing and referral to a hospital indicated by the MESP, because it disrupts continuity of care for the patient, respect of the pyramidal organisation of healthcare services, and reduces income in their own system. |
| MUSECCO | - With the payment system of capitation, MUSECCO was not directly confronted with problems of over-prescription, over-billing and fraud. From the members’ point of view, the payment system contained costs: they received care at a low price while their effective feedback mechanism guaranteed perceived quality, “the slightest flaw is instantly reported”. Other, non-member, patients seem to cover the losses for the providers due to undervaluation of the MUSECCO contribution (see Contribution of MHO to quality?). There were no comments linking capitation with cost-containment or with prescription behaviour. |
| UMUSAC | - Monthly validation corrects any invoicing that is not in accordance with the contract. - The UMUSAC health centre was created to promote cost containment and rational prescription. How this influenced other providers is not clear. |

**M3—Guarding over the long voice route: Schemes link communities with politically more voiced groups or hold government or other external actors accountable to regulate the health system**

**Creation of a platform for dialogue between stakeholders**

| MUSOSA | - MHO meets providers individually. - Maybe a platform for discussion in the making: Seeking assistance to calculate the current cost of the care package, the MHO approached the district health authorities. They studied average fees in the contracted health facilities and observed the need to promote rational prescription. They asked to be involved in the elaboration and signing of new contracts with the providers, based on the calculated fees. |
| --- | --- |
| Goma overall | - Meeting between CGAT, MHOs, health authorities and providers every three months. Discussion points are members’ complaints, providers’ complaints, the clauses of the contract especially the prescription of generic medicines, recurring problems with invoicing, sometimes case discussions. - The meetings are appreciated by all partners. - In 2016, the number of meetings was reduced to 1 or 2 due to budget constraints. |
| KLA | - See Goma |
| MUSSRA | - See Goma |
| South Kivu overall | - CAMS has one-on-one meetings with the providers once a year, when renewing the contract. - On December 23th 2015, CAMS organised a meeting with MHOs and providers, and had an initial discussion about a permanent framework for dialogue. But they would not be able to finance it. - Topics for discussion are the tariffs, the payment method and quality of care, which are the purpose of the partnership. |
| Nyantende | - The MHO sought the involvement of the health zone in its dealings with the health centres. Currently the health zone closely follows the cooperation and helps in problem solving. - The MHO organises regular meetings with health zone and providers. - The MHO is invited in the monthly meetings of the management committee of the health zone. - However, decisions made in common agreement are then not carried out. |
| Walungu | - The MHO manager participates to the monthly meetings of the management committee of the health zone. The officer in charge promotes cooperation with the health centres, but most are reluctant. |
| Kinshasa overall |  |
| Lisanga | - Three-monthly meetings with providers are appreciated by all partners. |
| MESP | - The medical advisors meet providers individually. There is no cooperation with health authorities beyond the initial negotiation of tariffs and contracts. |
| MUSECCO | - Meetings between MHO management and BDOM coordination 4 times per year. - MHO leaders are regularly invited to the monthly meetings of providers of the BDOM network. This is where preoccupations of MHO and providers are discussed. - This platform remains within the Catholic Church – MUSECCO was an initiative of the teachers of catholic schools. - Little contact with health authorities. |
| UMUSAC | - MHO meets providers individually. |

**Support organisation**

| MUSOSA | - Receives little assistance from the CGAT residing in Beni (59 km) since 2014 |
| --- | --- |
| Goma overall | - CGAT since 2012: coordinator, accountant, medical advisor, community mobiliser. |
| KLA | - CGAT provides technical support and close follow-up since 2014. |
| MUSSRA | - CGAT provides technical support and close follow-up since 2015. |
| South Kivu overall | - CAMS was created in 2000 as a department within the BDOM. - Team of 7: 1 coordinator, 2 medical advisors, 1 financial manager, 1 secretary, 2 field supervisors. - In 2015, CAMS became a separate entity under direct authority of the diocese. - In November 2015, the Network of MHOs (*réseau des mutuelles de santé communautaires de Bukavu* (REMUSACO)) was created. All MHOs are represented in the general assembly. The CAMS team became its executive secretariat, accountable to the general assembly. |
| Nyantende | - CAMS provides technical support and close follow-up since 2001 |
| Walungu | - Technical assistance by CAMS is problematic compared to other MHOs. This is attributed to the fact that CAMS is an organisation of the Catholic Church, whereas the hospital and the health zone of Walungu are not. |
| Kinshasa overall | CGAT:   - Created in May 2010. It was an initiative of the PNPMS, the international partners and the religious leaders. - Mission: to provide technical support to existing MHOs and starting initiatives; to organise them in unions and federations, and to integrate MHOs into the health financing system. - 16 MHOs in Kinshasa - Contracting, controlling quality and respect of norms and regulations is the role of the CGAT, not the MHO itself. MHOs mainly deal with members. |
| Lisanga | See Kinshasa overall. |
| MESP | - Technical support from the PNPMS for the initial study of the average fees for services. - Financial support from the Ministry of education for the preparations. - Substantial Government financial support. - Exchange with Belgian mutualities. |
| MUSECCO | - Technical support: ILO/STEP from feasibility study in 1999 until 2011. - One-time financial support: BTC, GTZ, CGAT for sensitisation, purchase of a plot - Training: PNPMS and the ILO/STEP - Current monitoring and technical support: PNPMS, PNCNS (National Health Accounts – *Programme National des Comptes Nationaux de la Santé*) and CGAT. |
| UMUSAC | UMUSAC was created in 2010 with the aim to coordinate and provide support to a network of MHOs, currently 12. The coordination team counts 7 persons. UMUSAC is in turn a branch of the Christian Workers Movement (MOCC). |

**Cooperation with other organisations and institutions**

| MUSOSA | - During preparation, cooperation was sought with CAMS in Bukavu and in Butembo with BDOM, Caritas, protestant churches, health zone, and managers of MHOs that ceased to exist. - Financial and some technical support from a Belgian MHO since 2010. - No cooperation with the health zone; starting cooperation with the health district   Options for cooperation:   - Cooperation with MHOs is mentioned in the action plans of provincial and local health authorities. - Cooperation proposed by the Health Services Purchasing Fund (*Le Fonds d’Achat des Services de Santé* (FASS): since 2008, FASS manages funds provided by international donors to improve access to health care services in rural North Kivu. Services are purchased at highly subsidised rates if quality conditions are met by contracted providers. FASS suggest that MHOs manage membership and members’ contribution, but delegate purchasing to FASS. Fixed fees and contracts were negotiated in the context of the European Development Fund (EDF) project. However, this option only concerns rural areas. - Urban actors point at negative consequences of the EDF project:   - “We manage to provide caesarean section at 120 $. In the EDF project, the patient pays 16 $, the project 180 $. When the project ceases, those physicians will be used to the high fees.”   - “Patients from the city seek care in the rural areas where services are subsidised.” |
| --- | --- |
| Goma overall | - The management committee of the CGAT is constituted by members representing 7 organisations among which: Mecreco, an important mutual credit organisation, Caritas, the most prominent protestant church (ECC: *l’Eglise du Christ du Congo*), the enterprise Asrames, the programme for women and environment, the international organisation ULB Cooperation.   Options for cooperation:   - There is a unit for MHOs at the level of the DPS. - Unicef supports this unit, but only cooperates with the government, not with the CGAT. - The organisation ULB Coopération cooperates with the provincial authorities to design a social health insurance system. |
| KLA | - Active cooperation with the health zone. - Following the CGAT guideline, the MHO office is provided by the government: it is situated in a government hospital. |
| MUSSRA | - See Goma |
| South Kivu overall | - In its relationship with other actors, the MHO network both benefits and suffers from its integration in the Catholic Church. On the one hand, the Catholic Church has undoubted authority as one of the most effective organisations. It is through their position within the diocese that the MHOs can have synergies with partners of the city or provincial administrations. On the other hand because of this connection, they are accused not to be “for all people”. The relatively greater effectiveness and financial means of the Catholic Church and of the MHO network cause frictions with the administrative authorities and agents. - “Before, all provincial government agents were members. This is no longer the case.”   External subsidies:   - Idjwi Sud: When Cordaid paid for the poorest, the MHO had sufficient cash to cover the care of all. Since Cordaid stopped subsidising, the number of members dropped - Kabare: Since IRC (International Rescue Committee) stopped subsidising, the number of members dropped - Burale : Amitiés Belgique – Bukavu ASBL (ABB) pays for management costs; Commission Justice et Paix (CDJP) paid the premium of about 2000-3000 young people, since they stopped subsidising, the number of members dropped. - GIZ pays management costs for Nyantende (200 $ per month), Nyangezi (100 $ per month), Luvungi (100 $ per month) et Mwana - Solidarité mondiale (WSM) pays 200 $ for the local manager of 23 MHOs, with the exception of Burale where ABB pays. - Mutualité chrétienne Hainaut Picardie (MCHP) pays the bonus (180 $) for 14 assistant managers   Effects international donors:   - Interventions are often not well adapted to the local context. International partners do not evaluate implementation and effects. - Fundamental dilemma:   - Subsidies are indispensable for the functioning of the MHOs and the health services.   - Chronic funding of healthcare by external donors erased the perception that health care is an expensive service that must be financed, and that the funds should be carefully managed and not misused. - “The notion is not there to use subsidies for empowerment so that tomorrow you can do without. Life reliance on Government subsidies can be accepted, not so life reliance on external donors.”   Options for cooperation:   - The Provincial Health Division appointed an MHO coordinator. - Many international organisations are active in the domain of healthcare. There is, however, very little cooperation among actors. |
| Nyantende | - “Health care providers will increase fees, overcharge, overprescribe when the MHO management is not strong. To be strong, a real cooperation with local authorities, religious authorities and school directors is necessary.” - Nyantende is the MHO with the largest membership in South Kivu (over 13000 in 2013). A prominent factor was the process of starting the MHO which involved widespread sensitisation and cooperation with all layers of society: first the community groups of the Catholic Church, then the political and administrative authorities, then other churches, especially the protestant church, development associations, NGOs, local leaders, local development groups, people coming to market and schools. The involvement of politico-administrative authorities was crucial. - Financial support from Maltezer from 2013 to 2015 to MHO and health zone, and subsidy for coverage of 1444 indigent (85 $ per person) - Currently financial support for MHO management from GIZ, and from MCHP and WSM via CAMS.   Options for cooperation:   - Current MHO leaders point at the necessity to cooperate with schools and political authorities. |
| Walungu | - There is little cooperation between actors in the area. The MHO management tries to bring actors together with little success. - All important actors were involved in the preparation, sensitisation and set-up of the MHO: the referral hospital and its partner the NGO FSKi, the health zone and health centres, administrative authorities of different levels including police and military, traditional leaders, religious authorities of various denominations. This cooperation did not continue once the MHO started operations. Respondents point at the importance of a history of free healthcare funded by external donors and the distrust about how these funds are used as underlying factor for the lack of cooperation:   - The majority of the population still believes healthcare is paid for by external donors. If hospital and MHO ask for people’s payment, most are convinced that MHO or providers use it for their own benefit.   - Administrative authorities discontinued cooperation once they understood that the MHO was not a project with substantial external subsidies. The MHO tries to regain their cooperation because of their huge influence in the community.   - Distrust about how money will be handled makes cooperation among other organisations and communities difficult.   External partners in the health zone:   - The referral hospital receives financial support from the NGOs FSKi and Louvain développement, and technical support from Medics without Vacations. - Several international NGOs support the health zone and/or various health centres. - The health zone receives technical support from the Public Health School of Bukavu through the project RIPSEC that promotes evidenced based decision-making.   Options for cooperation:   - The officer in charge of the health zone plans to improve cooperation between actors via the RIPSEC project. |
| Kinshasa overall | CGAT:   - Participation in advocacy, in promotion of legal framework through sustained cooperation with POMUCO, PNPMS and the Department of social protection of the Ministry of employment, work and social protection. - Little cooperation with the health authorities. No involvement of the local health authorities apart from co-signing the contracts with providers. - Exchange with international organisations involved in similar programmes. - International backing of Belgian Christian Mutualities (CM) |
| Lisanga | See Kinshasa overall. |
| MESP | - No involvement of health zones. - Exchange with international organisations involved in similar programmes. |
| MUSECCO | - Collaboration with Catholic and Salvation Army networks - Close collaboration with PNPMS and PNCNS (*Programme National des Comptes Nationaux de la Santé*) - Recent sensitisation campaign with Ministry of Health, city governorate and CGAT in all communes of Kinshasa. |
| UMUSAC | - At the start of the programme: involvement of local authorities and community associations (within the MOCC movement; also with others) - Good cooperation with health authorities and providers. - The MHO participates to events such as vaccination campaigns, condom distribution, etc. - Technical and financial support from MOCC and the NGO WSM. - Cooperation within the Christian Workers Movement, nationally and internationally. |

**M4—Short voice route: Social care and emancipatory programs increase poor peoples’ confidence to negotiate directly with providers over the quality of care**

**Information of members**

| MUSOSA | - Members are well informed about all aspects of the functioning of the MHO. |
| --- | --- |
| Goma overall | - “Members do not easily comprehend the concept of paying for healthcare. Because of frequent international emergency interventions, they are convinced the international community will continue to cover health care expenses. Currently, this opinion is maintained by the substantial subsidisation of healthcare services in rural areas.” |
| KLA | - Members do not understand yet that contributions limit the realisation of their demands. - Members have not been well informed about the benefit package. They were told that the MHO would cover 90% and the patient 10%, but most are prescribed non-generic drugs that are only covered at 50%. This omission is now being corrected. However requesting generic medicines in the first place is not promoted by the MHO manager. - The concept of cost-containment is not understood by members and MHO manager. The measure proposed to restore financial solvency of the MHO is to reduce the financial coverage of services by the MHO. |
| MUSSRA | - Members do not understand how the MHO functions. Many seek personal financial benefits above all. “Before starting, everybody should have been better prepared. Who has been prepared? When? For how long? Who has prepared them? Have the hospitals been prepared? Even the Provincial Health Division has not been prepared. Where are the Public Health Schools that know about MHOs?” - Sensitisation for the second year took lessons learned after bankruptcy on board: the effects of over-prescription and fraud, the quality of generics, the need to contain costs. |
| South Kivu overall | - A fundamental misconception by members and the overall population overpowers people’s understanding of what MHOs are: People are convinced that the MHOs function with external donors subsidies; that any deficit will be covered by donors. They do not understand that health care expenses are paid with members’ contributions only. - Frequent complaints about drug stock-outs in the health facilities and the fact that members have to buy drugs at private pharmacies out-of-pocket. - Increasingly, members who paid their contributions but are denied access because of the insolvency of their MHO come up for their rights. |
| Nyantende | - Members were sufficiently informed until the recent reduction of the benefits package. |
| Walungu | - After a long period of free healthcare, people are not used yet to the notion of paying for health services. New members are given all information about MHO functioning, benefits’ package and financial coverage, but only fully understand this when they have to co-pay hospital services. - “We tell patients they need this care but it will not be covered by the MHO. Patients have to accept, because when it is the provider who says he has to take this medication, or he should do this or that, he can discuss but ultimately he still pays.” (a provider) |
| Kinshasa overall | Most members do not understand or adhere to the rules and spirit of the MHO. Many seek to exploit the MHO. Understanding of rational quality prescription is poor. |
| Lisanga | See Kinshasa overall. |
| MESP | - Members are sufficiently well informed. They have easy access to information via the delegates in the health facilities, also via internet and MHO office. |
| MUSECCO | - Members are well-informed about their rights and obligations. - A widely distributed brochure contains all relevant information. |
| UMUSAC | - Members are well-informed about their rights and obligations. |

**Feedback mechanism members -> MHO -> providers**

| MUSOSA | - Active feedback mechanism |
| --- | --- |
| Goma overall |  |
| KLA | - Active feedback mechanism from members to MHO, but little action taken by the MHO management |
| MUSSRA | - Active feedback mechanism, but little change by providers |
| South Kivu overall | - Complaints are transmitted to the medical advisors. The most frequent complaint is: “we were seen, but we were given a prescription to buy the medicines elsewhere”. Hospital directors are reminded that this is not as agreed, but changing practice would need more complex intervention. |
| Nyantende | - Active feedback mechanism, but little change by providers |
| Walungu | - Very active feedback and attempts to solve problems by the MHO management. Few solutions are found because of the complexity of the issues and the concerted action that should be taken by all partners involved. - Example of MHO feedback, in this case successful: The MHO observed that patients treated for malaria were several times readmitted with the same diagnosis. Because the MHO has to pay for each episode, the manager reported this to the medical advisor, the health zone and the hospital and asked whether something could be wrong with the treatment. The medical advisor did not intervene. The health zone confirmed the relapses. Bed nets were distributed to the population and malaria treatment at the first line was investigated. If further action was taken is not known by the MHO manager. |
| Kinshasa overall |  |
| Lisanga | Active feedback mechanism. The medical advisors are frequently contacted by patients by phone whenever they have a problem or question. CGAT responds to each individual problem. |
| MESP | - “Given the social context, medical doctors will not listen to patients. But they discuss with the medical advisors when they have to request approval for certain investigations and treatments. Patients are better protected because they are backed by an organisation capable of intervening.” - Active feedback mechanism: delegates in health facilities, easy access to the MHO office to discuss problems, exchange between medical advisors and health facilities in case of problems. |
| MUSECCO | - Active feedback mechanism: members report incidents by phone or by filing a complaint at the MHO office; MHO leaders report and discuss the issues during regular meetings with providers and BDOM. - “The slightest flaw is instantly reported”, but complaints are addressed via the MHO and not directly between provider and patient. |
| UMUSAC | - Active feedback mechanism. Providers pay attention to the criticisms and claims of the members, channelled by the medical officer. It improves services. |

**Presence of MHO delegate in health facilities**

| MUSOSA | - No. |
| --- | --- |
| Goma overall |  |
| KLA | - No. |
| MUSSRA | - All actors advocate for the presence of an MHO agent in the health facilities, whose role would be to orient patients, listen to complaints, solve problems, detect fraud, supervise reception, check which services are effectively provided, cooperate with the medical advisor for validation of invoices, to be the interface between hospital and member and show members that the MHO cares. |
| South Kivu overall |  |
| Nyantende | - MHO manager visits hospitalised members every day. - MHO leaders suggest that there should be a CAMS delegate based in the hospital to control whether services invoiced were actually provided. |
| Walungu | - No. |
| Kinshasa overall |  |
| Lisanga | - No. |
| MESP | - MESP delegates in health facilities follow the reception and treatment of members. Most delegate supervise two health facilities. They control entitlement and whether services are provided according to the contract. - “The patient is no longer isolated, no longer passively undergoes services. His rights are better respected.” |
| MUSECCO | - No. |
| UMUSAC | - No, with the exception of UMUSAC’s own health centre. |

**Members’ control over decision-making in the scheme**

| MUSOSA | - 4 meetings of the general assembly per year. - Decisions are made by the general assembly, after presentation of the issues by the board of administrators, composed of 10 competent and active members, and thorough discussion. Members feel involved. |
| --- | --- |
| Goma overall |  |
| KLA | - There is an annual meeting of the general assembly attended by about 100 members. Statues and Rules and Regulations are not yet finalised. Members vote and to adopt the decisions proposed by the management. |
| MUSSRA | - A special meeting of the general assembly was held to inform members that the MHO was bankrupt. Nearly 500 members attended, showing their genuine concern. They decided to interrupt activities for the remaining two months and start again next year on a more sustainable basis. |
| South Kivu overall | - Accountability to members is seen by the CAMS team as the first mechanism to control misuse of funds that “is a fact of our society and therefore also of the MHO system”. Overall, members do not understand the basic financial concepts underlying health insurance. Their decision-making is not informed. - Most decisions are made by CAMS for all MHO’s (contracts and negotiation of fees, management procedures). MHOs decide the amount of members’ contribution. |
| Nyantende | - 2 meetings of the general assembly per year with up to 700 members. Board of administrators and control committee are regularly renewed through elections; accountability to members is transparent. “The voice of the members is heard”. - The MHO report to CAMS, who gives technical support. CAMS standardised management procedures for all MHOs and the medical advisor decides what is included in the benefits package for all MHOs of the network. Nevertheless the MHO leaders claim their “MHO is independent because they decide on members’ contribution and MHO activities. CAMS proposes solutions to problems, but the MHO decides.” - The MHO leaders appreciate the evolution in the relationship with CAMS. Since the creation of the network of MHOs (REMUSACO), they observe real accountability of CAMS to the general assembly of representatives of the 23 MHOs. This change profoundly influenced the capacity and willingness of MHO leaders to voice their questions about CAMS management. |
| Walungu | - Decision-making is with members: contribution, benefits’ package, MHO’s financial coverage. However, CAMS provides payment of the MHO management, which limits ability to challenge their decision-making. The creation of REMUSACO changes this relationship and the MHOs now have more say. - 2 meetings of the general assembly per year, with relatively poor attendance (60 representatives of households for latest meeting). People are not used to participate in decision-making concerning healthcare or paying for it. Concerning affiliation to the MHO, they will follow the community leaders. The latest meeting of the general assembly was the first one where members understood that it is their MHO, that the management is accountable to them and that their decision-making counts. - “We did not understand how the equation members’ contribution/expenses works. Now we can explain it to the members and decision-making will be more informed.” (MHO management) |
| Kinshasa overall |  |
| Lisanga | - 2 meetings of the general assembly per year - Latest meeting of the GA: 213 / 1151 members present; Important decisions were: Finalising statutes and rules and regulations; Limitation in coverage: from 6th episode/year, the patient pays OOP of 40% instead of 10%. - Although the CGAT act as overall decision-maker, the general assembly votes all decisions. - Internal audits every term; transparent accountancy accessible at any time |
| MESP | - General assembly of 35 people: 17 are delegates of the 10 teachers’ unions, 17 are prominent persons in the education sector, + the president of the board of administrators. |
| MUSECCO | - Very active members’ participation in decision-making and daily life of the MHO. - During a recent extraordinary meeting to revise the statutes of the MHO, 817 members participated. |
| UMUSAC | - Active members’ participation in line with the objectives of the Christian Workers’ Movement. - Decision-making by members in the General Assembly, but implementation does not always follow. |

**Capacity of the MHO, as an association of members, to promote change**

Elements included in the formulation of the question:

- change the interactions between healthcare users and providers
- influence care quality
- social changes in power relationships in the community, beyond access to health care
- Is the voice of the MHO heard?

| MUSOSA | - “The MHO does not have the authority to influence care quality and pricing.” - “Also the BDOM has lost its capacity to control care quality and tariffs, since it stopped providing medicines to the health facilities of its network.” - Controlling care quality, prescription and fees is the role of the health authorities. - “At national level, POMUCO could lobby more easily for rational prescription, or for training of physicians on the subject.” |
| --- | --- |
| Goma overall | - Small and slow improvement of providers’ prescription and billing because of very active control of the medical advisor. |
| KLA | - Any change is the result of CGAT interventions. |
| MUSSRA | - The MHO manager explains that his social position does not allow him to challenge medical doctors about over-charging. - Even the hospital director, who is generalist, cannot convince specialists to prescribe generic drugs. It is the health authorities who should enforce treatment guidelines. - “You need to be a doctor to discuss with doctors.” |
| South Kivu overall | - The ‘capacity of the MHO to promote change’ was interpreted by most respondents in terms of cost recovery for the hospitals: “where the MHOs are well managed and there is a good relationship with the health facilities, there is a positive effect of the MHO on hospitals: the number of patients who run away without paying decreases”. - “Social change may be an objective, but in practice, the MHOs are looked at in terms of paying a premium and gaining access to health care.” - “The fact that people put money aside for the eventual risk of healthcare expenses is a profound change. This new attitude is an important social progress.” - A profound change of power relationships within the network of MHOs is underway since the position of CAMS changed from manager to executive secretariat of the network of MHOs (REMUSACO), accountable to the general assembly of MHO leaders. During the latest meeting, the MHO leaders found issue with the financial report and challenged it. This change was induced by the new organisational structure of the MHO movement. - “In our dealings with healthcare providers, the providers have the upper hand. Even the CAMS team has limited influence. For example, in the case of an MHO that ran out of money in the month of June, the in-charge of a health centre refused to provide care if the MHO would not pay its debts first. We asked if they could provide care and the MHO would pay when they could. But the in-charge refused: the MHO has to honour its debts before members are accepted again. This situation of indebtedness puts the provider in a position of power.” |
| Nyantende | Negotiation between MHO and providers:   - The negotiation of hospital tariffs is done by the medical advisor. For the health centres, tariffs are set by the health zone. The contract is signed between the MHO and the providers, but negotiations are done by the CAMS, the hospital management and the health zone. It is a standardised CAMS contract. The issue of care quality is discussed by the medial advisor. The president of the MHO is present in all discussions.   The MHO does not manage to change prescription and invoicing of providers:   - The MHO management regularly confronts the hospital management, proof at hand, about over-charging and invoicing of services that were not provided, but nothing changes. - Their argument that the large MHO is the hospital’s principal client, and that harm done to the MHO will ultimately harm the hospital has no weight in the discussion. - “The hospital management promises change, even during the meetings of the general assembly, but the same problems continue in practice.” - “When we point at specific actions of specific hospital staff, the hospital management will always protect their personnel, even when we can show evidence.” - “When we challenge specific cases where invoiced services were not provided, they will tell us to mind our own business. We have to leave it there, and pay.” - “We report these cases to CAMS, who reports to the BDOM. We have never seen a BDOM representative who comes to verify facts.” - “Where money is concerned, it is as if we become enemies.” - "Whenever we have meetings with the hospital, we get along, they listen to us, we agree, but then nothing changes. Compared to them, and given social hierarchy in the DRC, we are powerless.”   Social hierarchy:   - “A hospital in-charge is between a rock and a hard place. On the one hand there is the BDOM, the diocese, with their objectives, and on the other hand the hospital needs the means to provide care. Towards higher management, the hospital does not have the liberty to propose what they would want. In the RDC, you do not argue with your chef. Even if your argument is logically sound, your superior will not accept it and you can be taxed a rebel.” - “But now that the archbishop is president of REMUSACO, the voice of the MHOs and the members may be heard.” |
| Walungu | - Each action for improvement needs cooperation between hospital, health zone and health centres, MHO and CAMS. The MHO does not manage to promote agreement between partners. - For example: the annual renewal of the contract was not yet signed because of (1) disagreement on tariffs and arrears to the hospital, and (2) the decision to change the MHO contribution from 80% to 60% of healthcare expenses, adopted in all MHOs, was not yet formalised. - Members are still discovering the functioning and the roles of the MHO. |
| Kinshasa overall |  |
| Lisanga | - The CGAT medical advisors manage to revise the invoice downwards, but do not manage to influence prescription. Involvement of the health authorities is needed to promote change. - Regular discussion between medical advisors and providers improves members’ reception, overall appreciation of the MHO, and may in the long term promote change. - No influence in larger hospitals. |
| MESP | - Manages to promote change in health centres, medical centres and smaller hospitals. Their main asset is sufficient financial resources to intervene effectively: “The main obstacles are: (1) the absence of the regulator: it is the role of the health authorities to control care quality and fees; (2) the providers’ perception that an MHO has money to spare; and (3) fraud. To intervene, we have the following advantages: - We have the backing of our minister, who obtained government subsidies for the MHO. You need the backing of a strong authority. - Compulsory membership with premium retention from salaries also secures the MHO’s income. - These resources give the MHO the opportunity to engage personnel that is capable of finding solutions for all problems.” - Has the capacity to negotiate tariffs and conditions with the BDOM, Salvation Army, provincial authorities and private medical centres. “We are careful to obtain favourable tariffs without affecting quality”. - The BDOM makes concessions with which they do not agree (disruption of pyramidal primary healthcare structure because of MHO selection of first line and referral health facilities and outsourcing). “We have to accept because otherwise we would risk to lose a large population. This is abuse of power (« *Là c’est du chantage.*»).” - Does not manage to influence care quality and affordability in the large hospitals: “We suffer from the absence of the regulator in negotiations with large health structures.” |
| MUSECCO | - MUSECCO, then MUSECKIN, was one of the first and most influential MHOs of the country and has as such promoted change. Its activities were widely known nationally and internationally. Within the DRC many other groups of teachers followed their example and started their own MHO. Currently its influence has substantially decreased, mainly because of the creation of MESP, also because of decreased technical and financial capacity. - Via the BDOM, MUSECCO still has capacity to influence providers’ behaviour towards its members (e.g. continued care despite payment that does not cover the costs of the provider). |
| UMUSAC | - The influence of the MHO lies in the fact that providers want to attract and retain a group of clients. This implies that they will try to satisfy MHO members. |
