# Supplementary material for: Can mutual health organisations influence the quality and the affordability of healthcare provision? The case of the Democratic Republic of Congo
Source: PLoS One. 2020 Apr 16;15(4):e0231660. doi: 10.1371/journal.pone.0231660 (PMC7162613; doi:10.1371/journal.pone.0231660)
Supplement: Attachment — Submitted filename: Response to Reviewers Letter 28 February 2020.pdf [file pone.0231660.s003.pdf]

INSTITUTE OF TROPICAL MEDICINE ANTWERP

Antwerp 28th February 2020,

To the editorial board of PLOS ONE

Re: PONE-D-19-28775 Can mutual health organisations influence the quality and the affordability of healthcare provision? The case of the Democratic Republic of Congo

Dear Madam, Sir,

I acknowledge receipt of the mail of Agnes Pap, academic editor PLOS ONE, from Friday 28th of February 2020.

Two issues are raised in the mail.

First, the validation of the ORCiD iD. We thoroughly followed the instructions via the Editorial Manager account, and validated the following iD: https://orcid.org/0000-0002-1452-0088

Second, concerning the Funding Statement, we agree that you update it on our behalf to be: "This study is based on material from previous research, commissioned by POMUCO in 2016, and supported by the Belgian Directorate-general Development Cooperation and Humanitarian Aid in the context of the programme MASMUT (Micro-assurance santé/Mutuelles de santé)."

***

I hope we have adequately addressed the various comments of the editorial board, and hope that our paper can now be accepted for publication in PLOS ONE.

Best wishes,

Bart Criel

Bart Criel, MD, DTM&H, MSc, PhD
Professor, Department of Public Health
Institute of Tropical Medicine
Nationalestraat, 155, B-2000 Antwerp
bcriel@itg.be
Tel (landline): +32 (0)3 2476293
